# Prolonged intratumoral treatment with TLR7/8 agonist R848 regulates the tumor immune microenvironment resulting in enhanced antitumor activity

**DOI:** 10.1080/15384047.2026.2670801

**Published:** 2026-05-12

**Authors:** Yue Zhang, Xueyi Sun, Shixuan Cheng, Siyu Qian, Na Wan, Fengyun Xing, Hongwen Li, Hang Gu, Zeyuan Wang, Meng Dong, Zhenzhen Yang, Shaoxuan Wu, Mingzhi Zhang, Zhiqiang Han, Xudong Zhang, Huitao Liu, Qingjiang Chen

**Affiliations:** a Department of Oncology, The First Affiliated Hospital of Zhengzhou University, Zhengzhou, China; b Graduate School, Henan Medical University, Xinxiang, China; c Office of General Affairs, Henan Academy of Innovations in Medical Science, Zhengzhou, China; d Office of General Affairs, Zhengzhou Weirui Biotechnology Limited, Zhengzhou, China; e College of Advanced Interdisciplinary Science and Technology, Henan University of Technology, Zhengzhou, China

**Keywords:** TLR7/8 agonists, prolonged intratumoral injection pump, R848, tumor immunotherapy, OX40

## Abstract

**Background:**

Toll-like receptor 7/8 agonists (TLR7/8a), such as resiquimod (R848), are highly potent in activating dendritic cells and thus hold promise for T cell-mediated tumor immunotherapies. However, the short half-life of these small molecules in the lesion and the associated systemic immunotoxicity post-leakage of the drug into the circulation make their clinical application challenging.

**Materials:**

To overcome these shortcomings, we tested prolonged TLR7/8a therapy by intratumoral infusion of R848 for 25 h using a micropump to achieve durable therapeutic effects while minimizing the proinflammatory cytokine levels in the plasma post leakage of the drug into the circulation.

**Results:**

The results showed that prolonged immunotherapy with R848 (as low as 1 μg) significantly suppressed tumor growth (inhibition rates up to 98%, *p* < 0.01) in treated mice compared to control mice receiving regular intratumoral injection of R848. Higher levels of CD86^+^or CD11c^+^ D.C.s, CD4^+^/CD8^+^/OX40^+^ T cells, and cytokines (TNF-α/IFN-γ) were observed in the tumors and spleens of the mice in the treated group compared to the sham group (*p* < 0.05), indicating efficient activation of local and abscopal immunity by prolonged therapy with R848. Furthermore, the R848 functional concentration assay demonstrated that the micropump prolonged the treatment time of R848 drugs in tumors and reduced the requirement for higher doses, enhancing safety.

**Conclusion:**

Taken together, this study provides new insights into TLR7/8a immunotherapy for improved clinical performance, with potential benefits for patients with superficial tumors amenable to prolonged intratumoral infusion via micropump.

## 1. Introduction

The immune system constantly recognizes and clears tumor cells to maintain health. However, tumor cells can evade recognition and elimination by the immune system through various mechanisms known as tumor immune escape. Tumor immunotherapy eliminates tumors by restarting and maintaining the tumor-immune cycle and restoring the body's antitumor-immune response.^1^ Representative therapies include immune checkpoint inhibitors, cancer vaccines and T cell therapies such as chimeric antigen receptor T-cell immunotherapy (CAR-T).^2^^,^^3^

Innate immunity identifies viral and microbial markers by using pattern recognition receptors (PRRs). One of the PRRs families is toll-like receptors (TLRs), which function as a bridge between innate and acquired immunity.^4^ The TLR7/8 agonist (TLR7/8a), represented by resiquimod (R848), has been studied as an antitumoral immunomodulator in preclinical studies^5^ and as a vaccine adjuvant for cancer immunotherapy in clinical trials, activating human adaptive immunity.^6^ TLR7/8a activates TLR7/8 to act on dendritic cells (D.C.s) to promote the maturation of D.C.s.^7^ The secretion of the cytokines IL-6, IL-12, and TNF-α, and the chemokines IL-8, MIP-1α, and MCP-1 is increased.^8^^,^^9^ T cell antigen presentation is activated in the local tumor immune microenvironment. R848 recruits CD4^+^/CD8^+^ T cells and reduces the immune escape of regulatory T cells (Tregs). However, TLR7/8a is a small-molecule adjuvant that lacks specific cell tropism. Intravenous injection of TLR7/8a may lead to systemic immune system hyperactivity, and consequent cytokine release syndrome (CRS), which is pretty difficult to tolerate *in vivo*.^10^^,^^11^ Therefore, TLR7/8a is regarded as an effective adjuvant for local antitumor therapies.^12^^,^^13^

The severe side effects of TLR7/8a are a bottleneck in current clinical application.^14^ To minimize the toxicity caused by the elevated concentration of active TLR7/8a in circulation after drug administration, researchers have utilized various materials for packaging TLR7/8a, such as nanogels^15^ and heat-sensitive liposomes,^11^ to change the structure of TLR7/8a molecules and control their release. However, these methods involve complicated mechanisms that have not yet been fully verified or chemical structures that might not apply to every TLR7/8a molecule, limiting the clinical application of TLR7/8a-based therapy.

In this study, we established a novel TLR7/8a therapy by prolonged intratumoral infusion of TLR7/8a using a micropump to increase the half-life in situ by maintaining the drug concentration in the lesion at a minimum practical level for a prolonged duration while minimizing the leakage of the drug into the circulation, causing CRS. The results showed that this therapy could effectively inhibit tumor growth locally with local and abscopal immune activation efficiently, and had a lower minimum effective drug dose as well as lower toxicity compared to the treatment group using regular syringe injection. TLR7/8a therapy based on prolonged intratumoral injection has excellent potential for clinical transformation in tumor immunotherapy, particularly for superficial tumors.

## 2. Materials and methods

### 2.1. Cell lines

The CT-26 murine colon cancer cell line and RMA murine T-cell lymphoma cell line were purchased from OriCell (Cat. # M1-0101) and WheLab (Cat. # C2233). CT-26 cells were cultured in DMEM cell culture medium(Gibco, Cat. # C11995500BT) and RMA cells were cultured in RPMI-1640 cell culture medium (Gibco, Cat. # C11875500BT) supplemented with 10% fetal bovine serum (FBS, Gibco, Cat. # 10099141C) and 1% penicillin‒streptomycin (Gibco, Cat. # 15140-122).

### 2.2. Animal studies

Female Balb/c and C57BL/6n mice (5‒6 weeks, ~20 g) were purchased from the Experimental Animal Center of Henan Province. Animal studies were conducted according to protocol No. 2021-KY-1173-001, which was approved by the Ethics Committee of Scientific Research and Clinical Trial of the First Affiliated Hospital of Zhengzhou University.

A total of 906 mice were used to investigate the *in vivo* biodistribution and antitumor efficacy of R848, which is critical for elucidating its pharmacological characteristics and mechanistic properties. All animals were maintained under specific pathogen-free (SPF) conditions in ventilated housing units with controlled environmental parameters: temperature, 24 °C ± 2 °C; humidity, 55%–65%; 12-h light/dark cycles; and ad libitum access to autoclaved food and water. The mice were divided by simple randomization. A double-blind design was applied throughout the experiment. Following completion of the experimental protocol, the subjects were humanely euthanized via cervical dislocation after induction of anesthesia with 1.5% isoflurane (v/v).

For the allograft murine tumor model, 108 BALB/c mice were inoculated with 5 × 10^5^ CT-26 cells, and 90 C57BL/6n mice were inoculated with RMA cells subcutaneously on the back. Mice with touchable tumorigenesis were selected and randomly divided into three groups (sham group, regular intratumoral injection group, and prolonged intratumoral injection group), with six or five mice in each group, for subsequent experiments.

To assess the dynamics of R848 levels in tumor and plasma following prolonged intratumoral injection, 708 tumor-bearing mice were treated with intratumoral injection of 50 μL phosphate buffer solution (PBS, Procell, Cat. # PB180327) for the sham group, with R848 (InvivoGen, Cat. # tlrl-r848) dissolved at different doses (200 ng, 1 μg, 5 μg and 10 μg) via a syringe (50 μL PBS) for the regular intratumoral injection group, or with R848 dissolved at different doses (200 ng, 1 μg, 5 μg and 10 μg) via a micropump system (InnoPump, China) (500 μL PBS) for prolonged intratumoral injection over 25 h.

Mice were given standard fodder and sterile drinking water, with weight and tumor size recorded every seven days. The mice were placed on a heating pad, and their temperatures were monitored closely. The tumor volume was calculated using the following formula: tumor volume (mm^3^) = (length × width × width)/2, and the tumor growth curve was drawn. The mice were euthanized by cervical dislocation on day 24 for sample collection (blood, tumor, spleen, heart, lung, liver, and kidney).

To assess the dynamics of R848 levels in tumors and plasma by therapy with prolonged intratumoral injection, the tumor-bearing BALB/c mice were divided into a regular intratumoral injection group and a prolonged intratumoral injection group and treated with three doses (1 μg/5 μg/10 μg) of R848 dissolved in PBS through intratumoral injection using a syringe (50 μL PBS) for regular intratumoral injection or a micropump system (500 μL PBS) for continuous injection for 25 h, respectively. The mice in the regular injection group were sacrificed 0, 5, 10, 15, 20, 30, and 60 min after injection. The mice in the prolonged injection group were sacrificed at 0 min, 5 min, 10 min, 20 min, 1 h, 3 h, 25 h, 25h + 5 min, 25 h + 10 min, 25 h + 15 min, 25 h + 20 min, 25 h + 30 min, 25 h + 60 min, *n* = 3 for each time point. The mice were euthanized at the planned time point, and samples (blood and tumor) were collected for subsequent analysis. To isolate PBMCs for subsequent experiments, whole blood was collected from the orbital sinus by removing the eyeballs.

### 2.3. Immunohistochemical (IHC) staining and multiplex IHC(mIHC) assay

Mouse tissue was fixed in 4% paraformaldehyde (Biosharp, Cat. # BL539A) for three days, dehydrated with gradient xylene (Macklin, Cat. # X820585) and ethyl alcohol (Macklin, Cat. # E809056), and embedded in paraffin for tissue sections of 4 µm thickness on slides. The slides were dewaxed in xylene I for 20 min, xylene II for 20 min, 100% ethyl alcohol for 5 min, 75% ethyl alcohol for 5 min, and distilled water for 5 min. After antigen retrieval by sodium citrate, the slides were incubated in 3% bovine serum albumin (BSA) for 50 min, followed by three washes in PBS. The slides were then incubated with the appropriate antibody concentration (described below) overnight at 4 °C, followed by three washes in PBS. The slides were incubated with horseradish peroxidase (HRP)-conjugated goat anti-rabbit IgG (H + L) secondary antibody (1:20000, Proteintech, Cat. # SA00001-2) at room temperature for 50 min, followed by three washes in PBS. The slides were stained with diaminobenzidine (DAB, Servicebio, Cat # G1212-200T), followed by three washes with PBS. Hematoxylin for nuclear staining. 100% ethylalcohol and xylene were used for dehydration. The sections were then sealed with neutral gum. The results were analyzed using the CaseViewer software (3D Histech, Hungary).

Antibody information: anti-mouse CD3 (1:200, Cell Signaling Technology, Cat. # 99940T), anti-mouse CD4 (1:200, Cell Signaling Technology, Cat. # 25229T), anti-mouse CD8 (1:500, Cell Signaling Technology, Cat. # 98941T), anti-mouse FoxP3 (1:500, Cell Signaling Technology, Cat. # 12653T), anti-mouse/human CD86 (1:250, Proteintech, Cat. # 13395-1-AP), anti-mouse/human Ki-67 (1:500, Servicebio, Cat. # GB11030), and HRP-conjugated goat anti-rabbit IgG (H + L) secondary antibodies (1:500, Servicebio, Cat. # GB23303).

The mIHC kits were used as indicated (Absin, Cat. # abs50030). The sections were incubated with CD11c antibody (1:200, Cell Signaling Technology, Cat. # 97585), F4/80 (1:300, Cell Signaling Technology, Cat. # 70076), CD86 (1:200, Cell Signaling Technology, Cat. # 19589), CD68 (1:200, Boster, Cat. # BA3638), and CD163 (1:200, abcam, Cat. # 182422). DAPI was used for nuclear staining. The results were analyzed using the CaseViewer software (3D Histech, Hungary).

### 2.4. Immunofluorescence (IF) staining

Fixation, deparaffinization, and hydration of the mouse tissue sections were performed using the same protocol described for IHC. After antigen retrieval using sodium citrate, the slides were incubated with 3% bovine serum albumin (BSA) for 50 min, followed by three washes in PBS. Then, CD4-FITC antibody was added to cover the tissues at an appropriate concentration (as described below) for overnight incubation at 4 °C, followed by three washes in PBS. OX40 or FoxP3 antibody was added to cover the tissues for another incubation overnight at 4 °C, followed by three washes in PBS. The slides were then incubated with Cy3-conjugated goat anti-rabbit IgG (H + L) at 4 °C for 50 min, followed by another three washes with PBS. The slides were finally stained with DAPI for 7 min, Sudan black for 3 min, rinsed with running water for 12 min, and sealed with Fluoro-Gel. Images were captured using an Axio Imager M2 microscope (Carl Zeiss Corporation, Germany).

Antibody information: CoraLite®488 anti-mouse CD4 (1:500, Proteintech, Cat. # CL488-65104), anti-mouse OX40 (1:100, Abcam, Cat. # Ab00110-6.1), anti-mouse FoxP3 (1:500, Cell Signaling Technology, Cat. # 12653T), and Cy3-conjugated goat anti-rabbit IgG (H + L) (1:500, Servicebio, Cat. # GB21303).

### 2.5. TUNEL apoptosis test

Fixation, deparaffinization, and hydration of the mouse tissue sections were performed using the same protocol described for IHC. The slides were then slightly dried and incubated at 37 °C for 20 min with protease K (1:1000, Servicebio, Cat. # G1234-1ML) to cover the tissue. After three washes in PBS, the tissues were permeabilized (Servicebio, Cat. # G1204-100ML) at room temperature for 20 min. The slides were covered with a reaction mixture (recombinant TdT enzyme: biotin-dUTP labeling mix: equilibration buffer = 1 µL: 5 µL: 50 µL) and incubated at 37 °C for 1 h. Following the reaction, the slides were washed three times in PBS, and then covered with streptavidin-HRP (1: 200) and incubated at 37 °C for 30 min. After three washes with PBS, the slides were stained with DAB, followed by three washes with PBS. Hematoxylin for nuclear staining. 100% ethylalcohol and xylene were used for dehydration. The slides were then sealed with neutral gum (Servicebio, Cat. # WG10004160).

### 2.6. Hematoxylin–eosin (HE) staining

Fixation, deparaffinization, and hydration of the mouse tissue sections were performed using the same protocol described for IHC. The slides were stained in hematoxylin solution for 5 min, dipped in water for a few seconds, 1% hydrochloric acid alcohol differentiated for 5 s, rinsed with 1% ammonia for 10 s, dipped in water for a few seconds, stained in eosin solution for 3 min, and finally dipped in water for a few seconds. The slides were examined under a microscope and sealed with neutral gum.

### 2.7. Flow cytometry(FCM)

To detect changes in the abscopal immune cell, spleen lymphocytes were isolated and made into a single-cell suspension. To detect changes in the abscopal immune cell, spleen lymphocytes were isolated and prepared into single-cell suspensions at 1 × 10^7^ cells/mL. 100 μL cell suspension were incubated with cell surface marker antibodies in the dark for 20 min at 4 °C and washed twice with cell staining buffer (PBS with 2% FBS) by centrifuge (400 × *g*, 5 min). According to the manufacturer's instructions, the cells were resuspended in 200 μL of cell staining buffer and analyzed using a FACS Calibur flow cytometer (BD Biosciences, NJ, USA).

All cell surface marker antibody information: anti-mouse CD45-PE (BioLegend, Cat. # 147711), anti-mouse CD3-APC-Cy7 (BioLegend, Cat. # 100221), anti-mouse CD4-PerCP (BioLegend, Cat. # 100434), anti-mouse CD8-Brilliant Violet 605 (BioLegend, Cat. # 100744), and anti-mouse OX40-Brilliant Violet 605 (BioLegend, Cat. # 119419).

### 2.8. Enzyme-linked immunosorbent assay (ELISA)

The tumors and plasma of 1 μg R848 injection mice in the three groups were collected to detect the difference in TNF-α/IFN-γ/IL-12 levels among the mouse groups by ELISA. The tumors were homogenized in PBS (1 mL PBS: 100 mg tissue) and centrifuged at 10,000 rpm for 10 min at 4 °C to obtain the tumor homogenate supernatant. Whole blood was collected using the eyeball removal method and centrifuged at 3000 rpm for 10 min at 4 °C. The supernatant was collected as the plasma. The tumor homogenate supernatant and plasma were subjected to TNF-α level detection using a mouse TNF-α ELISA kit (Proteintech, Cat. # KE10002) or a mouse TNF-α high-sensitivity ELISA kit (MULTI SCIENCES, Cat. # EK282HS), IFN-γ using the IFN-γ high-sensitivity ELISA kit (MULTI SCIENCES, Cat. # EK280HS), and IL-12 using the IL-12 ELISA kit (MLBIO Biotechnology, Cat. # ml037869) following the manufacturer’s protocols.

### 2.9. Blood biochemical assay

Mouse plasma was collected, and the samples were loaded on an automatic biochemistry analyzer (Chemray 800, Leidu Life Sciences, Shenzhen). The corresponding parameters were set, and the results were exported after detection.

### 2.10. Hemolytic assay

To collect normal mouse red blood cells, whole blood was collected from the orbital sinus by removing the eyeballs after euthanizing the mice. PBS was added as a negative control, and sterilized water was added as a positive control. The red blood cells of each group of mice were treated with normal saline. After centrifugation, the supernatant was taken to detect the absorbance value at an optical density (OD) 450 nm, measured by a Multiskan FC microplate reader (Thermo Scientific, Waltham, MA, USA). Finally, the hemolysis rate was calculated according to the following formula: hemolysis rate (%) = (OD_Experimental group_ − OD_Negtive control_)/(OD_Positive control_ − OD_Negtive control_) × 100%.

### 2.11. Detection of R848 concentration by PBMCs

Determining the R848 level in tumors and blood by LC–MS is time-consuming and might not reflect the functional R848 level. Thus, a standard curve was generated to estimate the R848 level in the tumor and blood by the level of TNF-α, a major downstream effector cytokine of the MyD88-dependent signaling cascade, after stimulation of TLR 7/8 by R848. Whole blood from untreated mice was used to acquire PBMC. Following the manufacturer's protocol, isolation was performed using a mouse peripheral blood lymphocyte separation kit (Solarbio, Cat. #P8620). The experimental process is illustrated in Figure 5.

1.5*10^5^ PBMC cells in 200 μL complete RPMI medium were cultured for 24 h at 37 °C with R848 concentrations of 2.5 ng/mL, 5 ng/mL, 10 ng/mL, 20 ng/mL, and 40 ng/mL in the medium. The TNF-α level in the PBMC supernatant was measured by ELISA. A linear standard curve of the R848 level versus the TNF-α level was constructed to calculate the R848 level in tumors or plasma by measuring the TNF-α level of PBMC stimulated by tumor homogenate supernatant or plasma.^16^ Every 1 g of tumor tissue was added to 1 mL of PBS to prepare the tumor homogenate. The tumor homogenate supernatant was obtained by centrifuging at 10,000 rpm for 10 min. The plasma was acquired by centrifuging at 3000 rpm for 10 min. At each time, 20 μL of tumor homogenate supernatant or plasma was added to the PBMSs to stimulate TNF-α secretion and detect the R848 concentration trend.

To calculate the R848 level, the total TNF-α level measured by the ELISA kit was denoted as TNF-α _(total)_. The background TNF-α levels in the tumor or plasma were measured as TNF-α_(tumor or plasma)_. TNF-α released by PBMCs without R848 stimulation is denoted as TNF-α_(blank)_. At the same time, the TNF-α level of PBMC stimulated by the tumor homogenate supernatant or plasma was marked as TNF-α_(R848)_. Finally, TNF-α_(R848)_ = TNF-α_(total)_ − TNF-α(tumor or plasma) − TNF-α_(blank)_ was used to calculate the R848 levels according to the standard curve equation.

### 2.12. Statistical analysis

Representative results of at least three independent experiments are presented as mean ± standard deviation. Statistical analysis was performed with one-way ANOVA or Student's test as indicated in the figure legends using SPSS 21.0 or GraphPad Prism 8.0. *p* < 0.05.

## 3. Results

### 3.1. Treatment by prolonged intratumoral infusion of R848 by micropump could potentially eradicate local tumors

**Figure 1.**
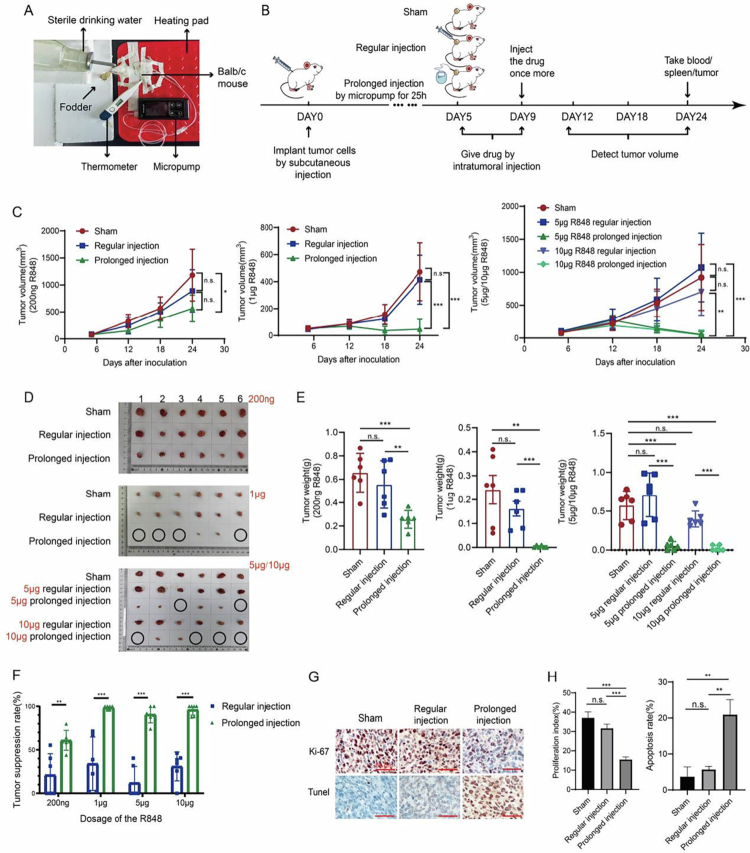
Assessment of the antitumor efficacy of TLR7/8a therapy with prolonged intratumoral injection of R848 by micropump system. (A) Structure of the prolonged intratumoral injection micropump in this study. (B) Scheme of animal experiments in murine allograft tumor models derived from CT-26 cells. (C–E) Improved therapeutic effects of treatment with prolonged intratumoral injection of R848 by micropump system versus treatment with regular intratumoral injection of R848 by syringe. (C) Tumor growth curve; (D) size of the isolated tumor; black circles indicate tumor disappearance. (E) Tumor weight; (F) Tumor suppressive rate of mice treated with 200 ng/1 μg/5 μg/10 μg R848 in the regular intratumoral injection group and prolonged intratumoral injection group. (G) Ki-67 staining and TUNEL assays detected the proliferation and apoptosis of tumor tissues from groups treated with 1 μg R848 by prolonged intratumoral injection, regular intratumoral injection, and sham. (H) Bar graph of the proliferation index and tunnel-positive rate of the cells derived from (G). All the data are expressed as mean ± standard deviation. *n* = 6 mice per group, 40×, scale bar = 50 μm, *p < 0.05, **p < 0.01, ***p < 0.001, n.s. = no significance.

To assess the TLR7/8a antitumor efficacy with prolonged intratumoral injection using a micropump system, we selected R848, a representative TLR7/8a molecule, and allograft murine tumor models derived from CT-26 murine colon cancer cells and RMA murine lymphoma cells. The tumor-bearing mice were randomly assigned to three groups: the sham group, the regular intratumoral injection group, and the prolonged intratumoral injection group (Figure 1A and B and S1A). R848 was tested at multiple doses, including 200 ng, 1 μg, 5 μg, and 10 μg. These results showed that tumor growth inhibition was significantly higher in all R848 dose tests in the prolonged intratumoral injection group than in the regular intratumoral injection and sham groups (Figure 1C–E and S1B–F). Although the tumor inhibition rate by 200 ng of R848 in the prolonged intratumoral injection group only achieved 60.9 ± 11.6% (*p* < 0.05, versus the regular intratumoral injection group), the rate by all other doses achieved a level higher than 90%, and *p* < 0.001 versus the regular intratumoral injection group in murine tumor models derived from CT-26 cells (Figure 1F). Strikingly, as shown in Figure 1D, some tumors in the prolonged intratumoral injection group were eradicated for all the dose tests except the dose test for 200 ng: 4/6 for the 1 μg group, 2/6 for the 5 μg group, and 4/6 for the 10 μg group. The therapeutic effects in the regular intratumoral injection group were not significantly different from those in the sham group in all the dose tests (Figure 1C–E).

Moreover, to rule out micropump operation affecting the experimental results, we supplemented the prolonged intratumoral injection with 1 μg PBS experiment as a control. The results showed that the regular and prolonged injection groups had no significant differences(*p* > 0.05). Prolonged injection of PBS and prolonged injection of R848 resulted in statistically significant differences (*p* < 0.01) (as shown in Figure S2A–C). We also observed the survival time of the four groups of mice. The survival time of the R848 prolonged-injection group of mice was significantly prolonged compared with that of the other three groups (Figures S1G and S2D). There was no statistical difference in the body weights of the mice in each group (Figures S1C and S2E).

Tumor cell proliferation and apoptosis were examined by Ki-67 staining and TUNEL assays of tumor tissues from the groups treated with 1 μg R848 and vehicle (Figure 1G). The results showed that treatment with 1 μg R848 by prolonged intratumoral injection significantly inhibited tumor cell proliferation and promoted apoptosis compared to treatment with regular intratumoral injection and sham, while significance versus sham was not achieved by the regular intratumoral injection group (Figure 1H). To detect immune cell changes in tumors in the prolonged intratumoral injection group, we repeated the experiment with 1 μg R848 to perform material detection, and the mice were sacrificed on day 18, when tumor shrinkage was expected (Figure 1G–H, *p* < 0.05). Taken together, our results suggest that treatment with prolonged intratumoral injection of R848 using a micropump system has significantly improved therapeutic effects compared with regular intratumoral injection using a syringe. More importantly, our results showed the therapeutic potential of TLR7/8a monotherapy in eradicating local tumors by prolonged intratumoral injection for drug delivery.

### 3.2. Prolonged intratumoral immunotherapy with R848 efficiently triggers immune activation in the local tumor microenvironment

**Figure 2.**
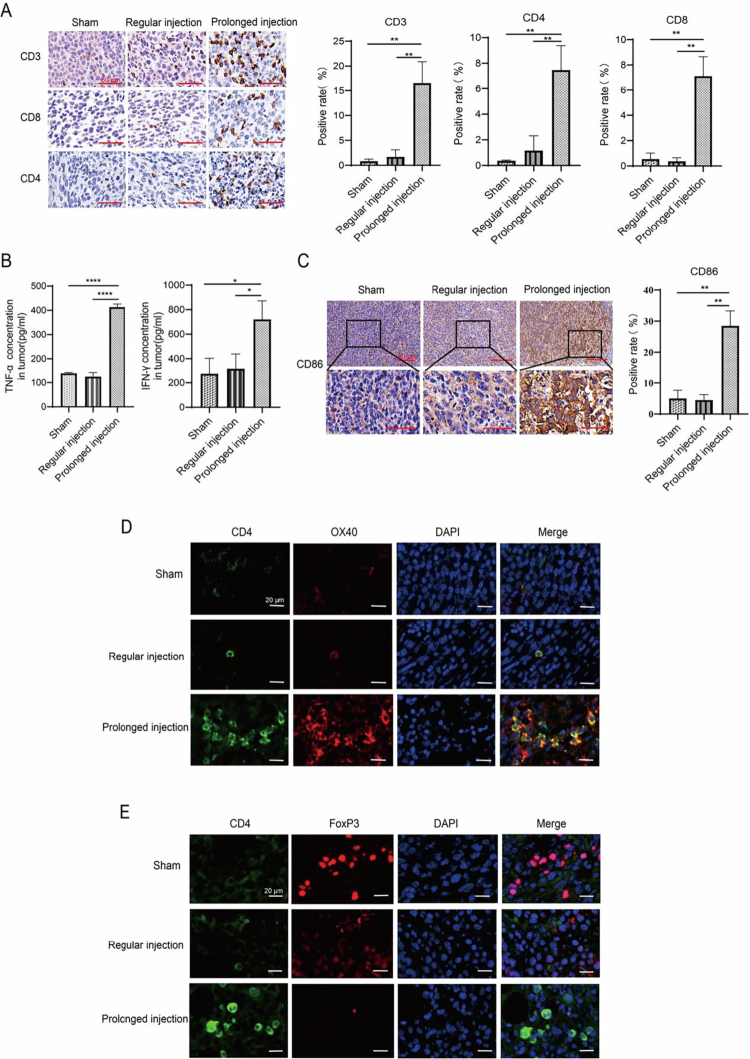
Prolonged intratumoral injection of 1 μg of R848 promotes remodeling of the tumor immune microenvironment. (A) Differential expression and positive rates of T cells in local tumor tissues from three groups: prolonged intratumoral injection, regular intratumoral injection, and sham (IHC). 40×, scale bar = 50 μm. (B) TNF-α/IFN-γ levels in tumors from the three groups (ELISA). (C) Differential expression and positive rate of CD86^+^ D.C.s in local tumor tissues from the three groups. 40×, scale bar = 50 μm. (D) Detection of CD4^+^ OX40^+^ T cell expression in three groups of tumor tissues (IF). 63×, scale bar = 20 μm. (E) Detection of CD4^+^ FoxP3^+^ T cell expression in the three groups of tumor tissues was tested by IF. 63×, scale bar = 20 μm. All the data are expressed as mean ± standard deviation. *p< 0.05, **p< 0.01, ****p< 0.0001.

TLR7/8a, like R848, is known to activate T cells by promoting the maturation and antigen presentation of D.C. To understand the mechanism responsible for the improved therapeutic effects of prolonged intratumoral injection, another animal experiment for 1 μg R848 testing was conducted, and tumors derived from CT-26 cells were collected for subsequent analysis. We first examined the expression of CD3^+^/CD4^+^/CD8^+^ T cells in the tumor tissues of the three groups (prolonged intratumoral injection, regular intratumoral injection, and sham), with a graph showing the positive staining rate of the cells. The results indicated that tumors treated with prolonged intratumoral injection had more active CD4^+^ and CD8^+^ T cells, indicating improved immune activation by prolonged intratumoral injection of R848 (Figure 2A, *p* < 0.05).

**Figure 3.**
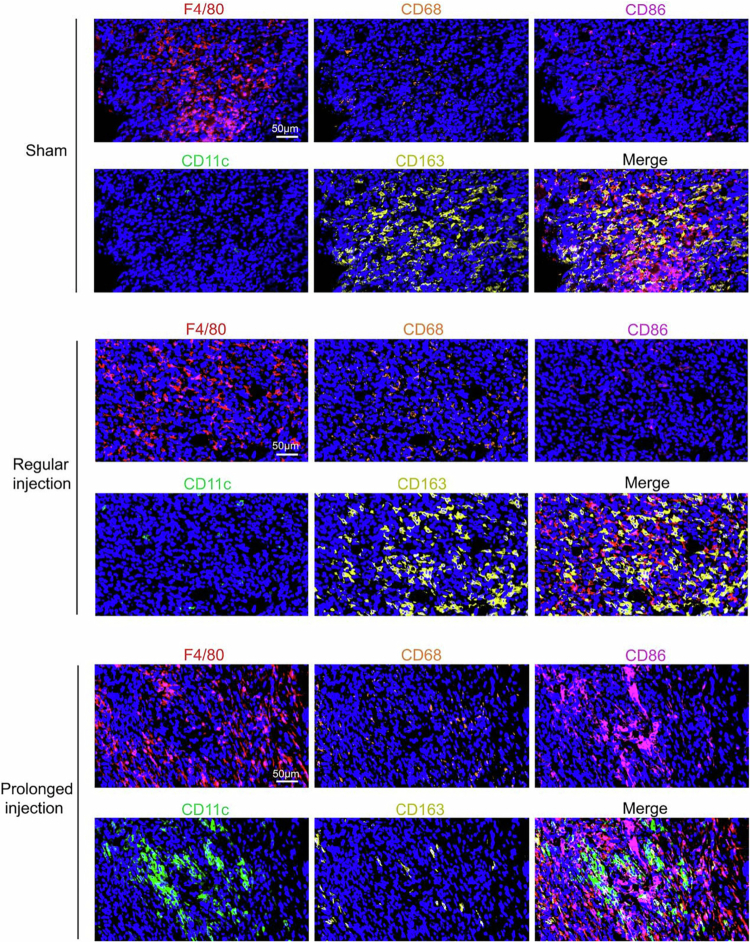
Prolonged intratumoral injection of 1 μg R848 altered macrophages expression in tumor microenvironment. Detection of immune cells expression in three groups of tumor tissues by mIHC. 63×, scale bar = 50 μm.

We next examined the TNF-α/IFN-γ levels in the tumors of the three groups by ELISA. As shown in Figure 2B, significantly elevated cytokine levels were observed in the prolonged intratumoral injection group (*p* < 0.05), whereas the difference was not significant between the regular intratumoral injection group and the sham group. We then examined active D.C.s by staining the tumor tissues with CD86 and CD11c antibodies. The results showed that CD86^+^ or CD11c^+^ D.C.s expression was highest in the prolonged intratumoral injection group, indicating improved activation of D.C.s by treatment with prolonged intratumoral injection of R848 (Figures 2C and 3).

Considering that OX40 is more highly expressed in activated CD4^+^ Teff cell populations and plays an antitumor role in immunotherapy,^17^^,^^18^ we measured the co-expression of CD4^+^OX40^+^ T cells in local tumors by IF. We found that OX40 highly colocalized with CD4 and was most abundant in the prolonged intratumoral injection group, suggesting that OX40 could be a potential target to synergize with R848 to enhance the therapeutic effects of CD4^+^ T cells when prolonged intratumoral injection was utilized (Figure 2D). Furthermore, we examined the abundance of T cells (Treg) by IF staining for FoxP3 and CD4. As shown in Figure 2E, while CD4 expression by prolonged intratumoral injection was higher than the other two, its FoxP3 expression was much lower than the rest. This result suggested that the expression of FoxP3^+^ Treg cells, which represent immune escape, was significantly suppressed.

Furthermore, we detected the expression of tumor-associated macrophages in the tumor microenvironment by mIHC. The expression of F4/80^+^ and CD68^+^ macrophages was similar in the three groups of tumor tissues derived from CT-26 cells. Compared with those in the sham and regular injection groups, the proportions of CD86^+^ M1 macrophages were increased and those of CD163^+^ M2 macrophages were reduced in the prolonged injection group (as shown in Figure 3).

The expression of CD3^+^/CD4^+^/CD8^+^ T cells was observed in the three groups of tumor tissues derived from RMA cells. These results confirm that RMA cells are T-cell lymphoma cells. In contrast to those in the sham and regular injection groups, the proportion of CD86^+^ M1 macrophages increased, while those of CD163^+^ M2 macrophages and FoxP3^+^ Treg cells were suppressed in the prolonged injection group (as shown in Figure S3).

### 3.3. Treatment by prolonged intratumoral immunotherapy with R848 activates abscopal immunity effectively and safely

**Figure 4.**
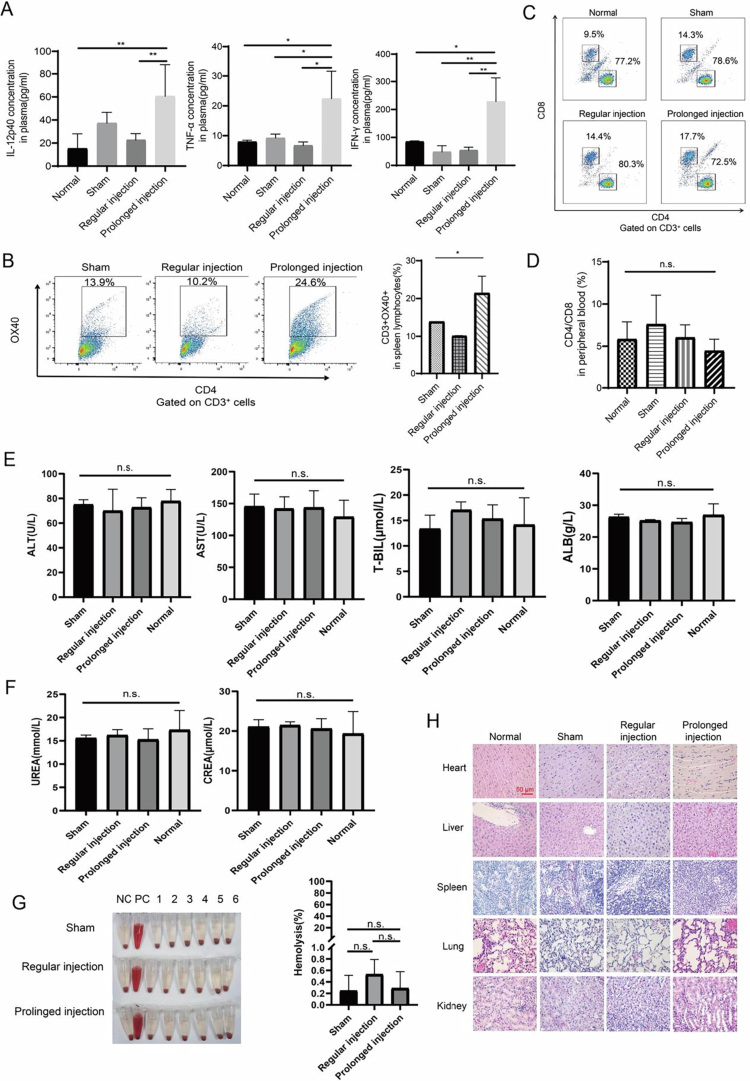
Prolonged intratumoral injection of 1  μg R848 activates the abscopal immunity with minor tissue toxicity. (A) Levels of TNF-α, IFN-γ, and IL-12 in plasma by ELISA after 1 μg of R848 was administered twice (*n* = 3 per group). (B) Flow cytometry to count CD3^+^OX40^+^ T cells in the spleen lymphocytes (*n* = 2 per group). (C) Comparison of CD4^+^/CD8^+^ T lymphocyte ratios in the peripheral blood of normal mice and the three groups of mice. (D) Bar graph of the CD4^+^/CD8^+^ T-lymphocyte ratios derived from (C). (E) and (F) Blood biochemical test results. ALT; Aspartate aminotransferase; AST; Total bilirubin; T-BIL; Albumin, ALB; Creatinine, CREA, creatinine. (G) Hemolytic experiments. NC; positive control; PC: negative control. (H) Hematoxylin and eosin (HE) staining to examine the tissue pathology of mice in the sham group, regular intratumoral injection group, and prolonged intratumoral injection group on day 24 compared to untreated mice. 10×, scale bar = 50 μm. All the data are expressed as mean ±  standard deviation. *p< 0.05, **p< 0.01, n.s. = no significance.

To examine whether the elevated immune activation by the controlled release of R848 is limited to the tumor, we examined cytokines (IL-12p40, TNF-α, and IFN-γ) and several immune biomarkers using samples collected from normal mice and mice from three groups: the sham group, the regular intratumoral injection group, and the prolonged intratumoral injection group. The ELISA results showed that the concentrations of IL-12p40, TNF-α, and IFN-γ were significantly increased in the prolonged intratumoral injection group compared to those in the other groups (Figure 4A, *p* < 0.05). Flow cytometry was performed to detect the expression of surface markers of spleen immune cells, and it was found that prolonged intratumoral injection of R848 not only activated T cells in tumors in situ and released cytokines into the peripheral blood but also stimulated increased expression of spleen OX40^+^ T cells (Figure 4B, *p* < 0.05). (Figure 4C and D, *p*< 0.05).

We also investigated whether prolonged intratumoral infusion of a micropump could inhibit distant tumor growth. We inoculated RMA murine lymphoma cells into the back and right flanks of the same mouse simultaneously. The tumors on the backs of the mice were administered R848 or PBS by prolonged or regular intratumoral injection (as shown in Figure S4A). The results showed that prolonged intratumoral injection of 1 μg of R848 significantly inhibited distant tumor growth (Figure S4B–D).

To assess the in vivo safety of 1 μg R848 controlled release, we performed flow cytometry to detect changes in the peripheral blood lymphocyte subsets of normal mice and three groups of mice. We found no statistically significant CD4^+^/CD8^+^ T-lymphocyte ratios between normal mice and the three groups of mice (Figure 4C and D, *p* > 0.05). Blood biochemical tests included hepatic function indices [alanine aminotransferase (ALT), aspartate aminotransferase (AST), total bilirubin (T-BIL), and albumin (ALB)] and renal function indices [UREA and creatinine (CREA)]. There were no significant differences among the three groups (Figure 4E and F, *p* > 0.05). Hemolytic experiments indicated that the hemolysis rates in all three groups were less than 1% and were not significantly different (Figure 4G, *p* > 0.05). HE staining was performed to compare the morphological changes in the heart, liver, spleen, lung, and kidney tissues of normal mice and the three groups of mice. The results indicated no significant morphological differences between the normal organs and the three groups of substantive mouse organs (Figure 4H). Similar results were observed in allograft murine tumor models derived from RMA lymphoma cells (Figure S5A and B).

### 3.4. Prolonged intratumoral release prolongs the therapeutic effects of R848 by keeping its in situ concentration above the functional concentration

Since the major clinical toxicity of R848 is caused by the abnormal upregulation of proinflammatory cytokines (e.g., TNF-α) in the blood following the leakage of large doses of R848 from the injection site into the blood, we attempted to assess the process of R848 leaking into the circulation from tumor tissue by analyzing the TNF-α secreted by PBMC stimulated using samples collected from mouse tumor tissue and plasma. This approach aimed to compare the differences in tumor immunity activation between regular and prolonged intratumoral injections.

**Figure 5.**
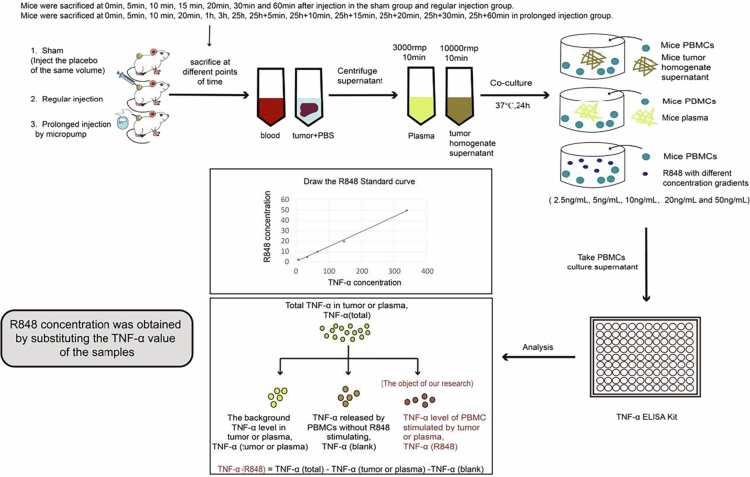
Illustration of the R848 concentration detection process in this experiment.

**Figure 6.**
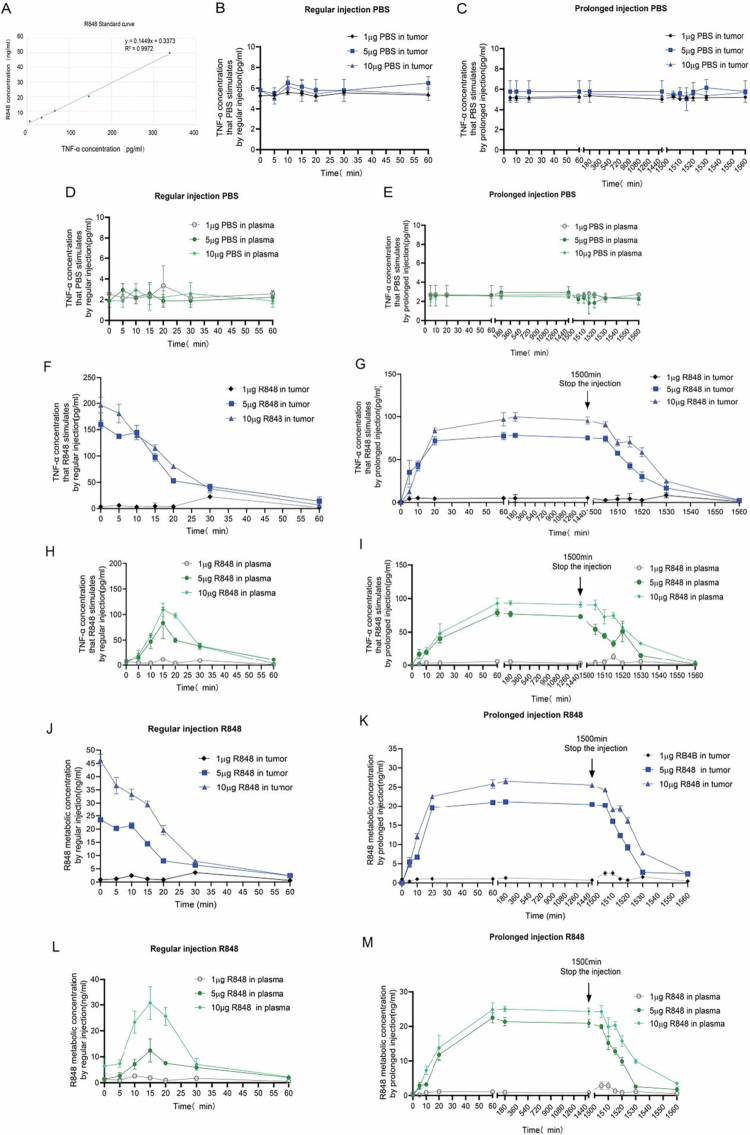
Treatment by the prolonged intratumoral injection extended the half-life of R848. (A) Standard curve of the R848 concentration. (B–E) PBS was regularly or prolonged injected into tumors, and we collected tumor tissue interstitial solution and plasma from the mice to stimulate TNF-α production in the PBMCs of normal mice. (F–I) Tumor tissue interstitial solution and plasma R848 stimulated TNF-α production in PBMCs from normal mice. (J–M) Metabolic concentration of R848 in the tumor and plasma. All data are expressed as the mean ± standard deviation (*n* = 3 mice per group).

Different concentrations of TLR7/8a (R848) stimulate normal lymphocytes to release different concentrations of TNF-α. We added 2.5 ng/mL, 5 ng/mL, 10 ng/mL, 20 ng/mL, and 50 ng/mL R848 to 1.5*10^5^ normal PBMCs per well. The concentration of TNF-α in the PBMCs supernatant was determined by ELISA assay after 24 h of R848 stimulation. The R848 concentration detection process in this experiment is shown in Figure 5, and we successfully mapped a linear R848 standard curve (R^2^ = 0.9972) (Figure 6A).

We selected doses of 1 μg, 5 μg, and 10 μg R848 to study the in vivo metabolic concentrations and pharmacodynamic stability of R848 in both the regular and prolonged intratumoral injection groups. TNF-α was released by PBMCs from normal BALB/c mice by collecting tumor tissue and plasma from mice treated at different time points. We specifically focused on the TNF-α concentration (TNF-α_R848_) released after tumor or plasma R848 stimulation of normal PBMCs (as shown in Figure 5). TNF-α_R848_ was substituted into the R848 standard curve equation to obtain the R848 concentration.

To explore whether the injection method intervenes in the stimulation of TNF-α, we injected PBS (1 μg, 5 μg, and 10 μg) in the way of regular intratumoral injection and prolonged intratumoral release at different time points. The tumors and plasma of PBS injection mice were collected for co-culture with normal mouse PBMCs. TNF-α levels in the culture supernatant (TNF-α total), tumors/plasma (TNF-α tumors/plasma), and mouse PBMCs (TNF-α blank) were detected. We calculated TNF-α PBS and compared the differences between the groups. TNF-α (PBS) = TNF-α(total) − TNF-α (tumors/plasma) − TNF-α (blank). Our results showed that the TNF-α concentration in the PBS group at different time points was constant. PBS stimulating TNF-α concentration in the tumor was 5–6 pg/mL, and in plasma was 2–3 pg/mL no matter of regular injection or prolonged release (Figure 6B–E).

The relative concentration trends of R848 at different time points were compared. After regular injection of 5 μg and 10 μg R848, the intratumoral drug concentrations decreased rapidly to below 10 ng/ml within 30 min, and the plasma drug concentrations decreased rapidly within 30 min after a brief increase of 15 min. However, with prolonged intratumoral injection of 5 μg and 10 μg R848, relatively effective concentrations of the intratumoral and plasma-retention drugs were maintained at approximately 25 h (1500 min) (Figure 6J‒M). No metabolic trend was detected in the 1 μg R848 injection group (including the regular and prolonged injection groups) (Figure 6F–M).

## 4. Discussion

For most drugs, increasing the dose does not result in better therapeutic benefits. Our research aimed to reduce the effective drug dose of TLR7/8a and achieve durable therapeutic effects. Previous research has reported that 10 μg is an effective dose of R848 in mouse models.^19^ In a mouse model of CT-26 in combination with A20 tumors, TLR agonists was 50 μg to activate the immune system in vivo based on the study of Levy’s group.^20^ Zhang et. al. found that 10 μg of R848 was injected into the tumor as a safe and effective dose using thermosensitive liposomes (TSL) as a delivery vehicle.^11^ Our group further reduced the dose of R848 to 1 μg, achieving a treatment efficacy that was almost equivalent to R848 at 10 μg. When the dose of R848 was further reduced to 200 ng, the therapeutic effect was significantly inferior to that of 1 μg. Thus, we investigated how the tumor microenvironment was modulated by prolonged intratumoral immunotherapy with R848 at a dose of 1 μg.

TLR7/8a delivers adjuvant-like signaling to antigen-presenting cells that express MHC I and/or MHC II molecules, where antigen peptides are present in CD8^+^/CD4^+^T cells.^21^ R848 activates CD86^+^ D.C.s and inhibits CD4^+^ Foxp3^+^ Treg-mediated immune escape.^22^^-^^24^ D.C.s stimulate OX40^+^ expression in CD4^+^ OX40^+^ T-cells.^25^ OX40 also inhibits Treg cell function and promotes Teff cell activation.^17^^,^^26^ Our research findings are consistent with the aforementioned mechanisms. Moreover, R848 primarily engages TLR8, triggering downstream signaling to enhance cytotoxic granule release and proinflammatory cytokine secretion. This activation potentiates NK-mediated lysis of virus-infected or malignant cells, bridging innate defense and adaptive priming.^27^^,^^28^ Notably, in depleted or exhausted T cells, R848 may partially reverse exhaustion by upregulating co-stimulatory molecules and restoring TCR-mediated signaling.^29^^,^^30^ Furthermore, investigating whether prolonged intratumoral TLR7/8a therapy induces durable immunological memory, which is capable of preventing tumor recurrence upon rechallenge, represents a critical next step for assessing its long-term therapeutic potential. In memory cells, R848 amplifies antigen-specific recall responses by promoting their proliferation, differentiation into effector cells, and cytokine production. It also enhances memory B cell antibody secretion, reinforcing long-term protective immunity.^31^^,^^32^ These underlying mechanisms were not addressed in our current study and warrant further validation in future research. In addition, we observed a higher expression of OX40 on the surface of splenic T lymphocytes in mice treated with prolonged intratumoral infusion than in those treated with regular injections. Considering that OX40 on T cells is a key therapeutic target for immune checkpoint regulation and has the function of modulating cytokine receptor signaling,^33^ we believe that the upregulation of OX40 may be explained by the release of proinflammatory cytokines (e.g., IL-12, TNF-α, and IFN-γ) released from tumors in situ into plasma and stimulating abscopal immune production.

In response to the failure of TLR7/8a (R848) in a regular intratumoral injection group to activate local immune production, we consider that R848 is a small-molecule drug in which low-dose R848 short exposure to tumor cells does not effectively activate in situ tumor immunity and is released into peripheral blood with rapid metabolic degradation.^34^ In this study, we used micropumps to control the dose and timing of the injection. This pump is very mature for clinical applications. In the field of oncotherapy, clinicians usually use chemotherapy to inject chemotherapy drugs, such as 5-fluorouracil.^35^ In our study, the translational applications of TLR7/8a may be promoted, in part, by altering the manner in which TLR7/8a was injected to effectively extend the retention of R848 in tumors treated with low doses. Furthermore, as reviewed by Gallio et al., imiquimod represents a safe and effective topical treatment for anal HSIL, particularly for perianal lesions. However, the response rates observed in intra-anal HSIL are substantially lower than those for perianal disease. This disparity is likely attributable to insufficient drug delivery efficiency and the establishment of an immunosuppressive tumor microenvironment within intra-anal lesions, which collectively limits therapeutic efficacy.^36^ Our proposed strategy of sustained intratumoral drug administration via a micropump offers an innovative approach to address these limitations. Mechanistically, imiquimod functions as a Toll-like receptor 7 (TLR7) agonist. Its suboptimal efficacy in the intra-anal mucosa may be explained by poor drug distribution, brief mucosal residence time, and inadequate local immune activation—particularly in anatomically complex regions such as the dentate line. Drawing on insights from studies of the R848 delivery device, an intra-anal drug delivery system capable of precise localization and sustained release could enable prolonged and enhanced innate immune activation at the target site. This approach may represent a key advancement in overcoming current therapeutic barriers. Collectively, these mechanistic insights highlight the potential of optimized local drug delivery strategies to improve the clinical efficacy of imiquimod for intra-anal HSIL.

To compare the differences in tumor immunity activation elicited by the two types of injections, we analyzed the correlation between the R848 concentration and TNF-α secretion by PBMCs upon R848 stimulation, employing an approach similar to that described by Gorden, K.K. et al.^16^ We found that TNF-α was linearly related to R848 with higher sensitivity (Figure 6A) and could be used to detect active R848 or its functional metabolites to induce TNF-α expression in PBMC cells. Although less accurate than the LC-MS-based method, detecting R848 using TNF-α-based functional testing would allow our results to align better with R848 toxicity in mice. Unfortunately, with samples from mice treated with R848 at 1 μg either by prolonged infusion or regular injection, we did not detect that the TNF-α levels induced were significantly higher than those in samples from sham mice (Figure 6B–M), suggesting that 1 μg R848 for mice treatment would be too low to be monitored by TNF-α-based functional detection. As such, we increased the dose of R848 to 5 μg and 10 μg, measured the functional level of R848, and observed significant TNF-α induction in both tumor tissue and plasma from mice treated with prolonged infusion, indicating the durable existence of R848 or its functional metabolites until the end or the 25th hour in the tumor tissue and plasma (Figure 6J–M). In comparison, in the mice treated with regular injection, the TNF-α induction level peaked in the beginning and rapidly declined in tumors with a peaked at approximately 15 min in the plasma, suggesting that R848 quickly moved outside from the injection site into the blood. It would be reasonable to conclude that the difference in antitumor efficacy between prolonged infusion and regular injection resulted from the difference in the drug concentration at the tumor site or the half-life of R848, which is about 20 min vs. 25 h for doses of R848 at both 5 μg and 10 μg, suggesting that prolonged infusion can physically increase the half-life of R848 at the tumor site for enhanced antitumor efficacy.

While our murine model demonstrated good tolerability for both intratumoral injection methods at the effective dose (1 µg R848), it is critical to note that murine models often underrepresent the severe systemic toxicities (e.g., cytokine release syndrome) observed with TLR7/8 agonists in clinical trials.^10^ The primary safety advantage of the micropump demonstrated here is the reduction in peak plasma drug levels compared to a bolus injection (Figure 6J–M), achieved by maintaining functional drug concentrations within the tumor over an extended period while minimizing leakage. This pharmacodynamic profile suggests the potential to mitigate systemic toxicity in clinical applications, where such toxicity is a major barrier. However, a definitive assessment of the micropump's ability to improve the therapeutic index by reducing clinical toxicity requires further evaluation in larger animal models and ultimately clinical trials.

Despite the superior performance observed with prolonged intratumoral infusion of R848 compared with regular intratumoral administration of the drug, there are several limitations of this treatment method. First, intratumoral administration is largely limited to superficial tumors. Although CT guidance may be feasible, it would be technically impossible or more difficult for prolonged intratumoral infusion than for regular intratumoral injection. Second, the mechanisms of abscopal immunity were not fully explored in this study, especially the mechanisms by which cytokines released from orthotopic tumors affect the expression of OX40 in spleen T lymphocytes. Furthermore, deeper immune mechanisms involving NK cells, memory cells, and depleted T cells require further exploration to facilitate the potential of their clinical translation.

## 5. Conclusion

Our study demonstrated that prolonged intratumoral immunotherapy with TLR7/8a improves therapeutic effects by modulating the tumor microenvironment for both local and abscopal immune activation. Considering the higher rate of tumor elimination observed, this study would potentially benefit patients with refractory superficial tumors where prolonged intratumoral infusion of TLR7/8a by a micropump is applicable. In addition, our research also provides a valuable reference for other experimental TLR7/8a and other cytotoxic drug developers to further improve therapeutic effects and safety.

## Supplementary Material

Supplementary material Supplementary Figures.docx

ARRIVE_guidelines_checklist.pdf Supplemental Material

## Data Availability

Data supporting the findings of this study are available from the corresponding author upon reasonable request.
