# Supplementary material for: Prolonged intratumoral treatment with TLR7/8 agonist R848 regulates the tumor immune microenvironment resulting in enhanced antitumor activity
Source: Cancer Biol Ther. 2026 May 12;27(1):2670801. doi: 10.1080/15384047.2026.2670801 (PMC13173998; doi:10.1080/15384047.2026.2670801)
Supplement: Supplementary material — Supplementary Figures.docx [file KCBT_A_2670801_SM3236.docx]

**Fig. S1**


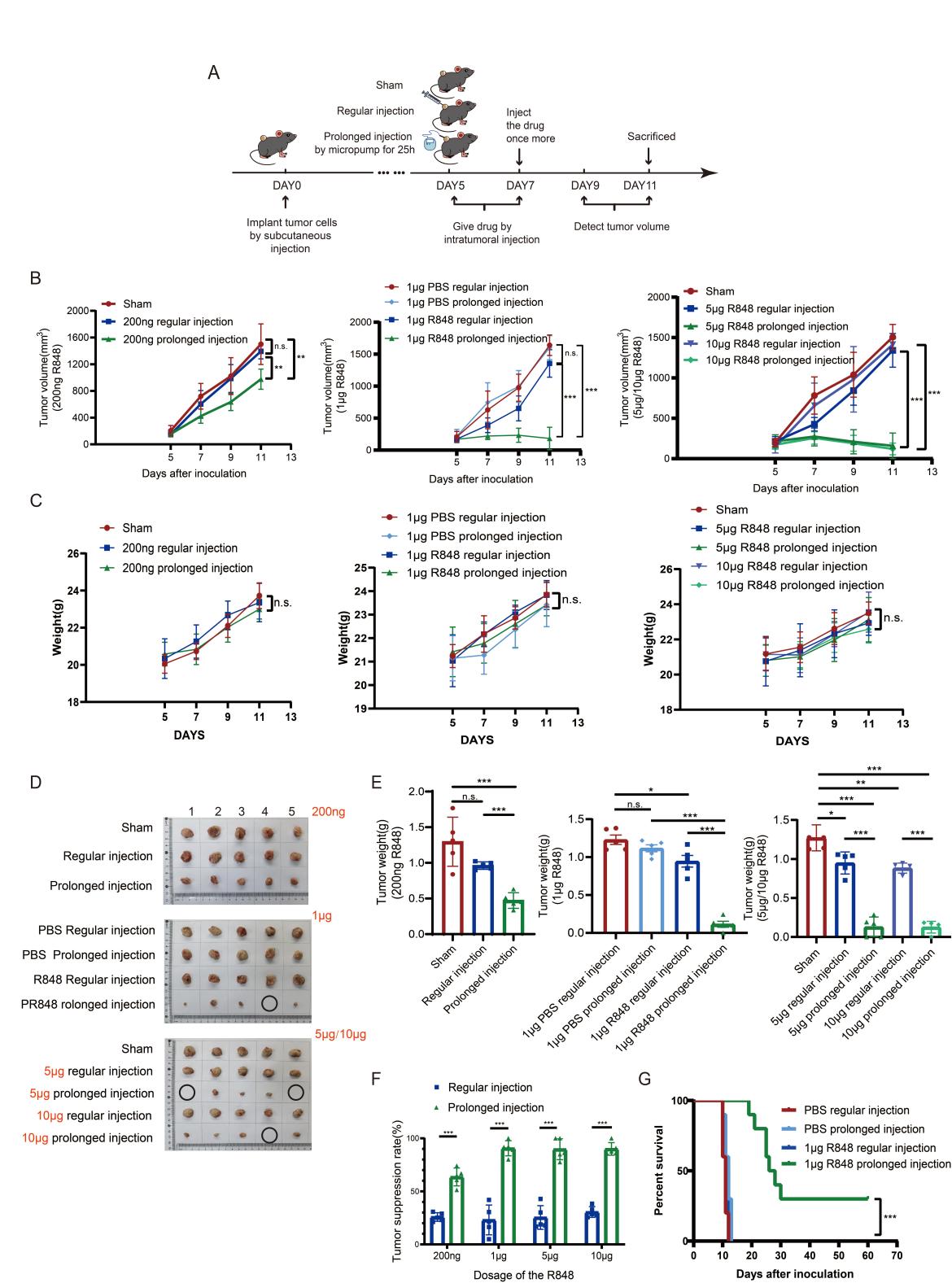


**Fig.S1 Drug efficacy tests of prolonged intratumoral injection R848 in allograft murine tumor models derived from the RMA cells.**

1. Scheme of the animal experiment. (B)Tumor growth curves. (C) Body weights of the mice in each group. (D) Size of isolated tumor: The black circles indicate tumor disappearance. (E) Tumor weight; (F) Tumor suppression rate of mice treated with 200ng/1μg/5μg/10μg R848 in the regular and prolonged injection groups. (G) Survival curves. All data are expressed as mean ± standard deviation. n=5 mice per group. **P*＜0.05, ***P*＜0.01, ****P*＜0.001，n.s.= no significance.

**Fig. S2**


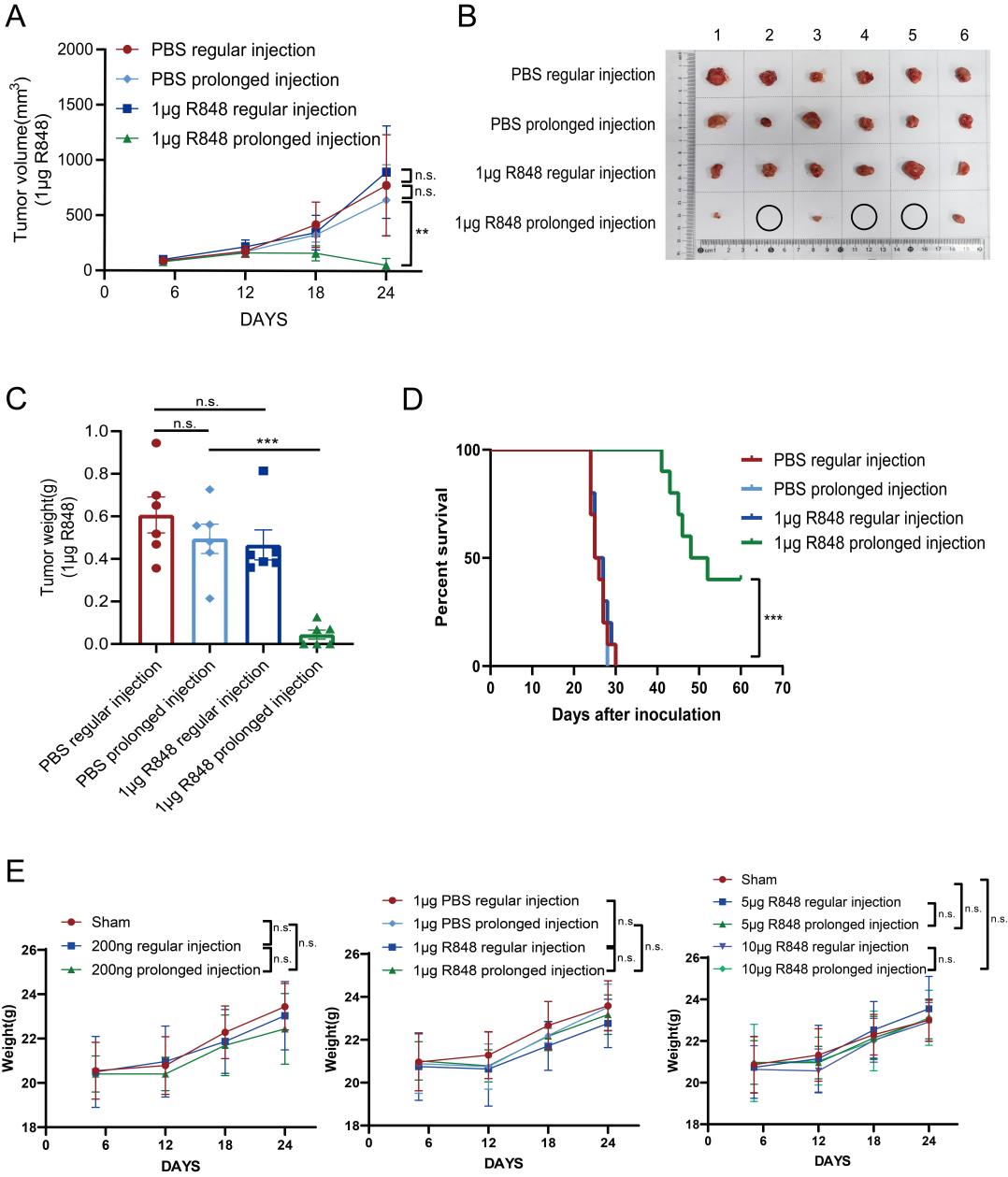


**Fig.S2 Prolonged injection of 1μg R848 by micropump system versus treatment with regular injection by syringe in allograft murine tumor models derived from the CT-26 cells.**

1. Size of isolated tumor. (B)Tumor growth curve: Black circles indicate the disappearance of tumors. (C)Tumor weight. (D) Survival curve. (E) Body weights of the mice in each group. ***P*＜0.01, ****P*＜0.001, n.s.= no significance.

**Fig. S3**


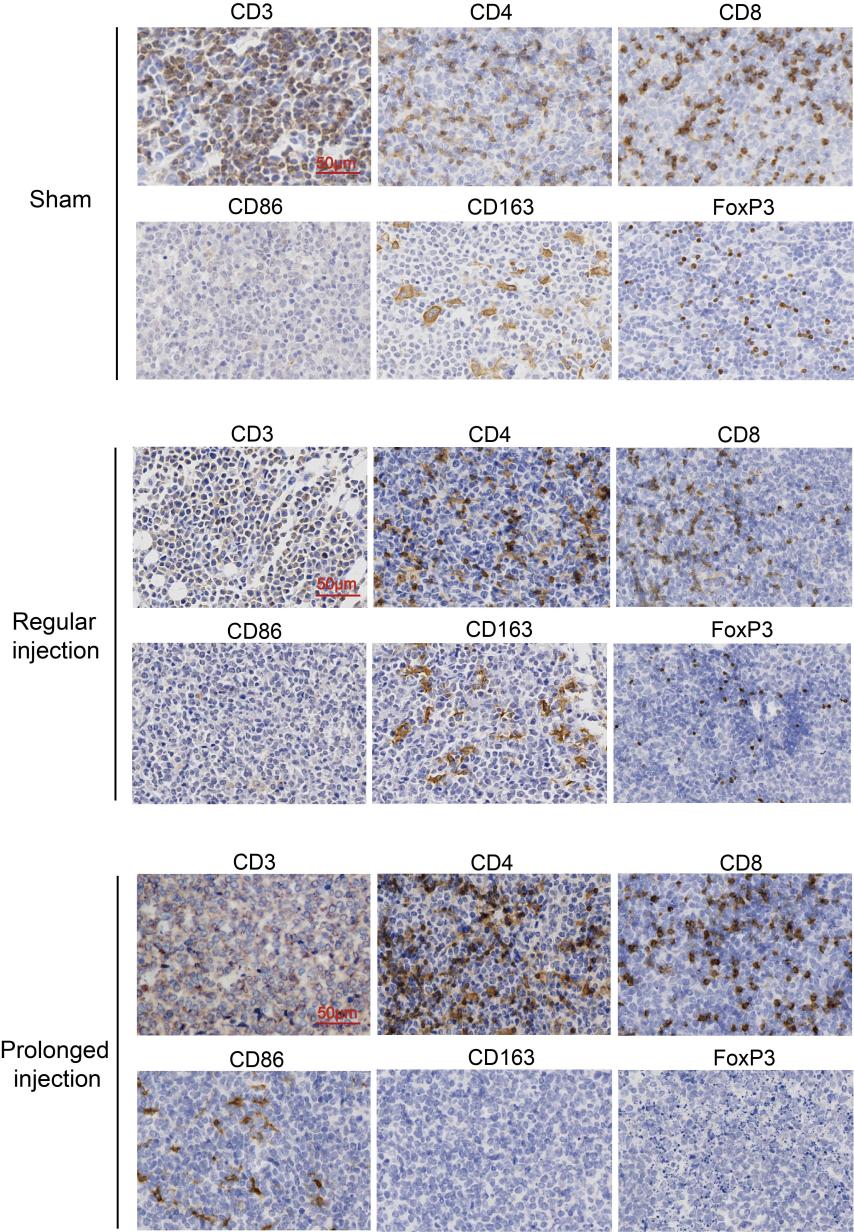


**Fig.S3 Prolonged injection of 1μg R848 promotes remodeling of the tumor microenvironment in allograft murine tumor models derived from the RMA cells.** 40×, scale bar=50μm.

**Fig. S4**


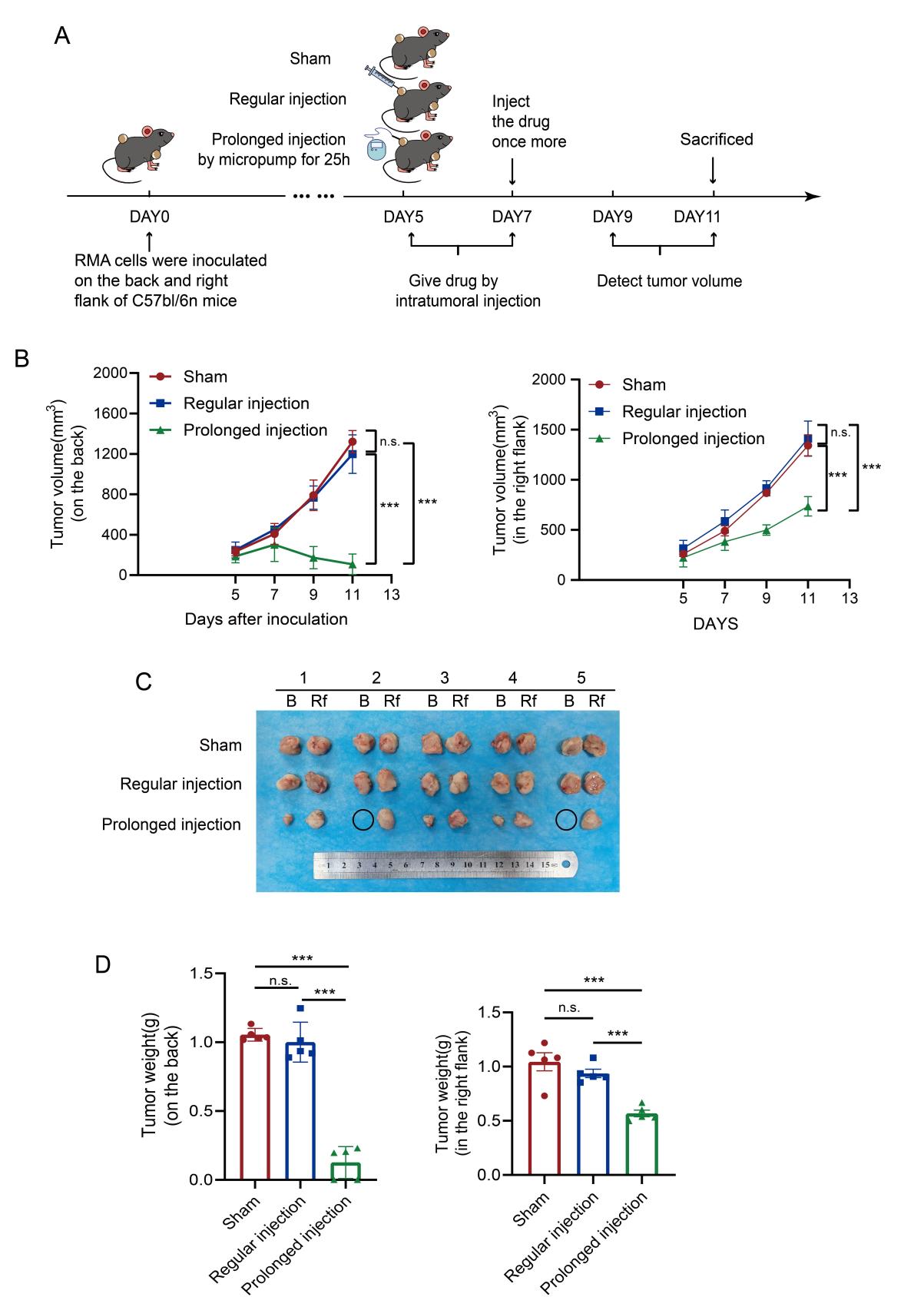


**Fig.S4 Prolonged intratumoral injection 1μg R848 inhibited the distant tumor growth.**

1. Scheme of the animal experiment. (B)Tumor growth curves. (C) Size of the isolated tumor. The black circles indicate the disappearance of the tumors; on the back, B; on the right flank, Rf. (D) Tumor weight. All data are expressed as mean ± standard deviation. n=5 mice per group. ****P*＜0.001，n.s.= no significance.

**Fig. S5**


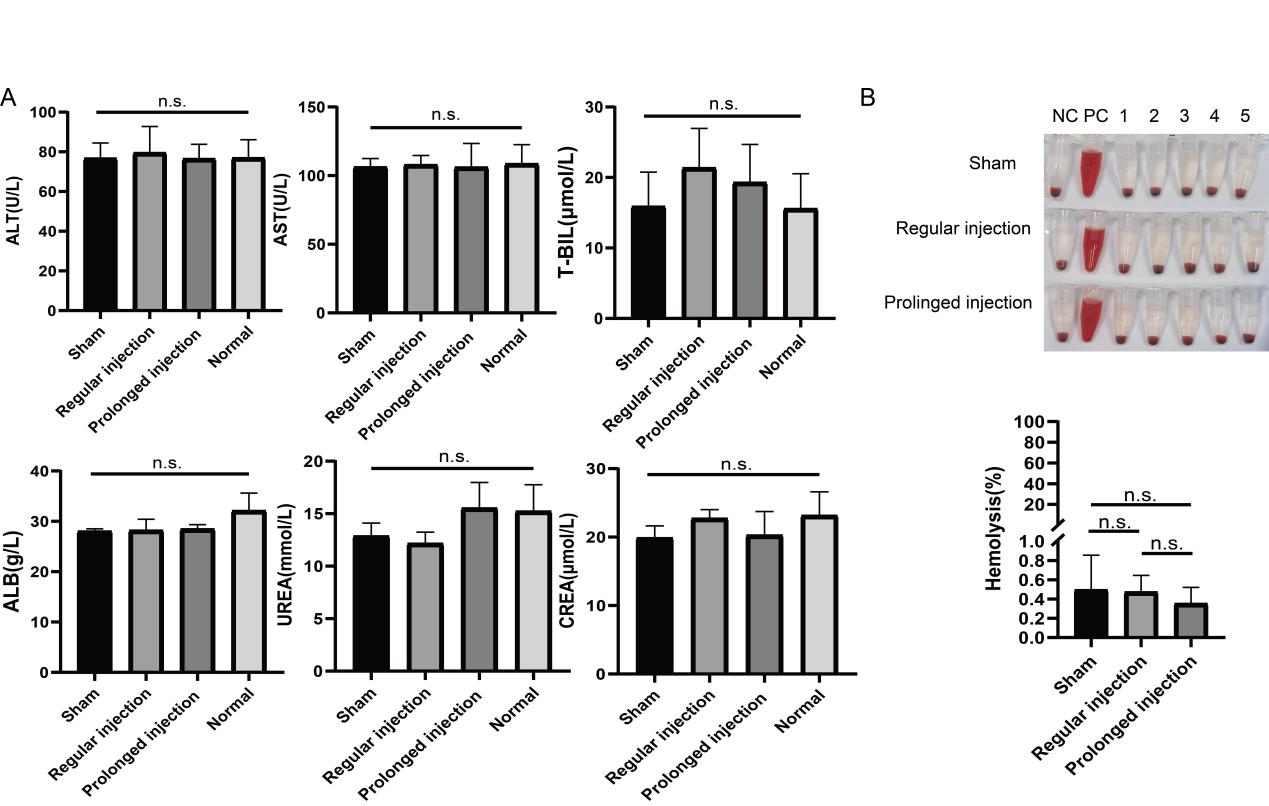


**Fig.S5 Prolonged intratumoral injection of R848 demonstrated good safety in allograft murine tumor models derived from the RMA cells.**

(A) Blood biochemical tests after 1μg R848 administered . (B) Hemolytic experiment. Negative control, NC; Positive control, PC. All data is expressed as mean ± standard deviation. n=5 mice per group. 10×, scale bar=50 μm. n.s.= no significance.
